# Evaluation of peripapillary choroidal distribution in children by enhanced depth imaging optical coherence tomography

**DOI:** 10.1186/s12886-018-0839-6

**Published:** 2018-07-13

**Authors:** Yi Zha, Jinfei Zhuang, Yixia Du, Jianqiu Cai, Haihua Zheng

**Affiliations:** 1 The 2nd Affiliated Hospital and Yuying Children’s Hospital of Wenzhou Medical University, Wenzhou, 325027 Zhejiang China; 2 Nanjing Children’s Hospital, Nanjing, Jiangsu 210000 People’s Republic of China

**Keywords:** Peripapillary choroidal thickness, Enhanced-depth imaging optical coherence tomography, Peripapillary retinal thickness, Children, Myopia

## Abstract

**Background:**

To evaluate the peripapillary choroidal thickness (PPCT) in Chinese children aged 6 to 12 years old and to analyze correlative factors.

**Methods:**

PPCT was measured with enhanced depth imaging optical coherence tomography (EDI-OCT) in 154 children (76 myopes and 78 emmetropia) aged 6 to 12 years, with spherical equivalent refractive errors between + 0.50 and − 5.50 diopters(D). Peripapillary choroidal imaging was performed using circular scans of a diameter of 3.4 mm around the optic disc. PPCT and the corresponding peripapillary retinal thickness (PPRT) were measured by EDI-OCT at nine positions: I, inferior; IN, inferonasal; IT, inferotemporal; N, nasal; T, temporal; S, superior; SN, superonasal; ST, superotemporal, and the Fovea Centralis.

**Results:**

The mean global PPCT was 165.80 ± 39.86 μm.The mean global PPRT was 101.47 ± 10.74 μm. The Inferior had the thinnest PPCT but the thickest PPRT (*p* < 0.001), while the Nasal had thickest PPCT but the thinnest PPRT (*p* < 0.001). Significant differences in RT between the myopic group and the emmetropic group were found at all positions except T, TS, S and the fovea. Myopic group had thinner choroidal thickness (CT) than that of emmetropic group at all measured positions. Choroidal thickness had negative relation with the corresponding retinal thickness, age and axial length.

**Conclusion:**

The peripapillary choroid was thicker nasally and thinner inferiorly, while the peripapillary retina was thickest inferiorly and thinnest nasally. Myopic group had thinner PPCT. The axial length was found to be negatively correlated to PPCT.

## Background

The choroidal blood flow is the highest of any tissue in the body to satisfy the normal metabolic demands of the outer retina [1]. The choroid provides metabolic support to the prelaminar portion of the optic nerve head [2, 3] and therefore plays an important role in many diseases related to optic disc such as glaucoma [4, 5]. Recent animal studies showed that the choroid also played an important role in the regulation of eye growth through the secretion of growth factors. Choroidal thickness was altered during the modulation of refractive status, which indicated its association with the development of children refractive errors [6–10].

With the advent of enhanced-depth imaging optical coherence tomography (EDI-OCT), it is possible to quantify the thickness and structure of the choroid in vivo. Most OCT studies have focused on choroid thickness at the fovea and evaluated its relationship with refractive error and axial length [11–13]. Only some studies measured peripapillary choroidal thickness (PPCT) [14–16] while very few studies focused on PPCT in pediatric population [17, 18]. The purpose of this study was to evaluate the choroid and the corresponding retinal thickness around the optic disc and their distribution pattern in healthy Chinese children aged between 6 and 12 years and to find possible link between choroid and other parameters.

## Methods

A total of 154 eyes from 154 Chinese children aged between 6 and 12 years in our hospital for physical examinations were enrolled in this study. Subjects were divided into two groups according to their spherical equivalent refractive error (SE): myopic (76, with SE between − 0.50D and − 5.50D) and emmetropic (78, with SE between ±0.50D). This prospective study was approved by the Institutional Medical Ethics Committee and adhered to the tenets of the Declaration of Helsinki. Informed written consent was obtained from all the participants and their parents.

The inclusion criteria for this study were normal visual acuity in both eyes of 20/20 or better, refractive errors of lower than ±6.0 diopters, astigmatism lower than 1.50 diopter, IOP below 21 mmHg. The exclusion criteria included any history of ocular disease, posterior staphyloma and tilted disc, ocular surgery, injury or any other systemic abnormalities such as vascular disease, hypertension, diabetes mellitus and family history of glaucoma. In addition, children with unclear OCT image or couldn’t cooperate with the examination were also excluded. All images were recorded by an experienced ophthalmologist. All examinations were performed between 10 and 11 AM to avoid diurnal variation. Two investigators, who were masked to judge whether the eye was amblyopic, measured the choroidal thicknesss. The final thickness for the analysis was calculated as the averaged value of the two investigators. The differences between the readings of the two investigators were found to be within 10% of the mean. All children recruited in our study completed a thorough ophthalmic examination including the non-cycloplegic refraction, best-corrected visual acuity (BCVA), IOP, slit lamp biomicroscopy, fundus examination and the axial length measured by partial optical coherence inferometry (IOLMaster; Carl Zeiss Meditec, Inc.).

### Optical coherence tomography imaging

**Fig. 1:**
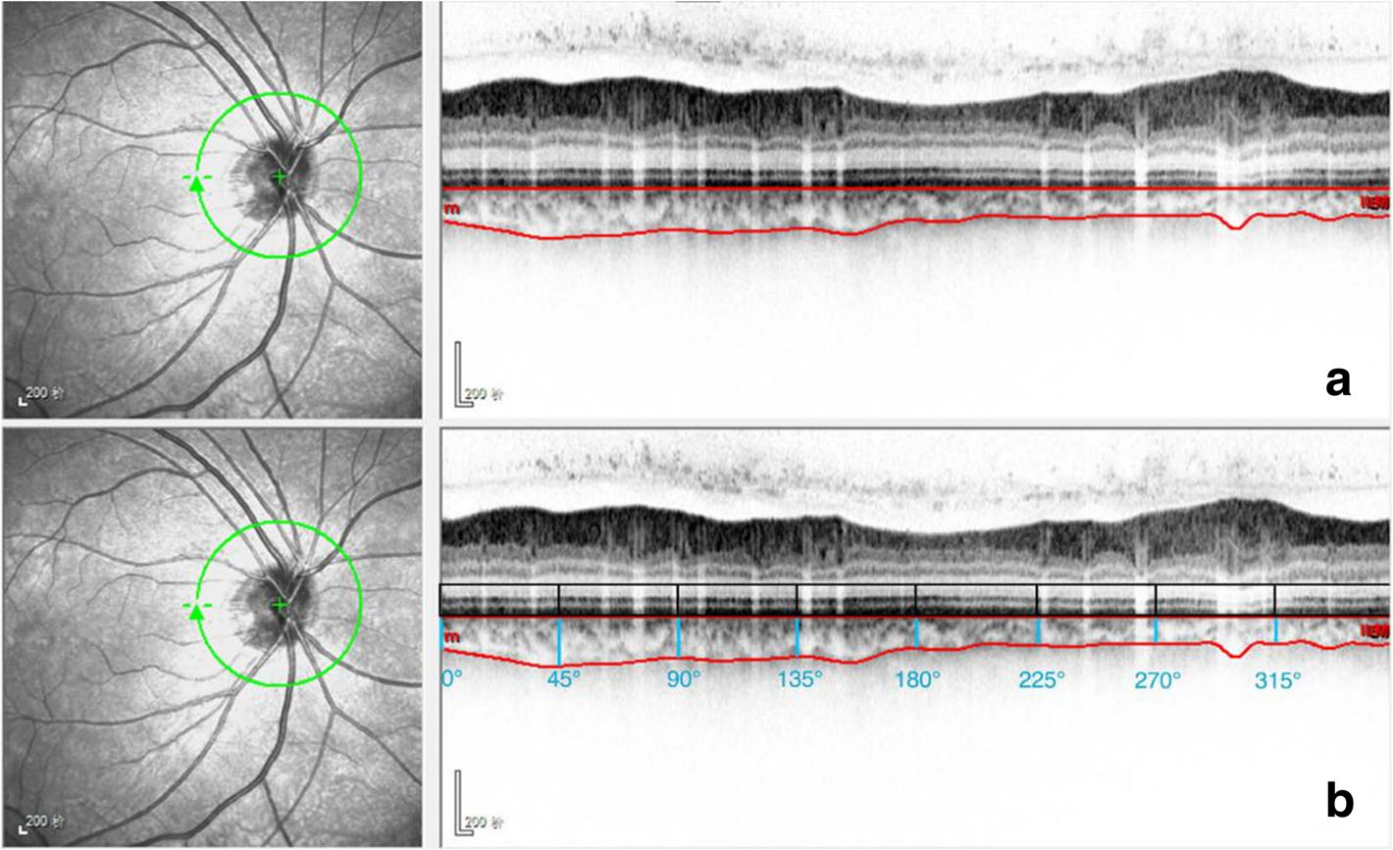
Peripapillary choroidal thickness measurement at eight locations. **a** Manual delineation of the outer and inner choroid borders using eye tracking software (Heidelberg Engineering). **b** Measurement of the peripapillary choroidal thickness at eight locations using image-processing software

In this study, the SD-OCT device (Heidelberg Eye Explorer v. 5.3; Heidelberg Engineering, Heidelberg, Germany) was used by the same investigator for measurment of all the children with undilated pupils. The images of OCT were taken in the conventional mode. We used a 360^0^, 3.4-mm diameter peripapillary circle scan (comprising 100 averaged scans and centered on the optic disc) for the peripapillary RNFL thickness. An ophthalmologist manually moved in a masked manner the segmentation line of the inner limiting membrane to the outer border of the choroid, which was defined as the hyper-reflective line of the inner surface of sclera, on the basis of files with segmentation of “Retinal” (with the upper line corresponding to the internal limiting membrane and the lower line corresponding to the retinal pigment epithelium/Bruch’s membrane complex). The images were then exported. Both peripapillary choroid thickness (PPCT) and peripapillary retinal thickness (PPRT) were measured at eight locations (temporal (T), superotemporal (TS), superior (S), superonasal (NS), nasal (N), inferonasal (NI), inferior (I), and inferotemporal (TI)) with each equidistant (45^0^) to the next location (Fig. 1). The measurements obtained from eight locations were averaged as global PPCT and global PPRT.

The subfoveal choroidal thickness (SFCT) and foveal retinal thickness (FRT) were measured as well. Only right eyes’ data of each participant was used for statistical analysis.

### Statistical analysis

SPSS software version 21.0 for Microsoft Windows (IBM Inc., Chicago, USA) was used for statistical analysis. All variables were checked with the Kolmogorov–Smirnov test. Because all of them were distributed normally, they were expressed as mean ± SD. Pearson correlation coefficients were calculated to evaluate relationships between PPCT, axial length, age and PPRT. Independent T test was used to compare the variables between the two groups. A value of *p* < 0.05 was considered to be statistically significant.

## Results

Of the total 154 children participated in this study, 92 were boys and 62 were girls. The average age of all the children was 8.96 ± 1.81y (range, 6-12y). The average SE was − 1.00 ± 1.31D (range, − 5.25D to + 0.25D). The average AL was 23.37 ± 2.85 mm (21.69 to 26.29 mm). No significant effect of gender was observed both in the CT and RT (both *p* > 0.05).The demographic characteristics of the children were shown in Table 1.

**Table 1:** Demographic characteristics of the participants

	Total	Myopia group	Emmetropic group
Age, year	8.96 ± 1.81	9.93 ± 1.85	8.06 ± 1.23
Sex, n (%)	154	74	80
Male	92	37	55
Female	62	37	25
Refractive error (D)	−1.00 ± 1.31	−2.08 ± 1.15	−0.00 ± 0.17
Axial length (mm)	23.37 ± 2.85	23.65 ± 4.06	23.12 ± 0.65

The mean global PPCT was 165.80 ± 39.86 μm. The mean global PPRT was 101.47 ± 10.74 μm. Tables 2 and 3 showed the CT and RT at different locations. Post hoc analysis utilizing least significant difference (LSD) - test demonstrated that the Inferior had the thinnest PPCT but the thickest PPRT (*p* < 0.001), while the Nasal had the thickest PPCT but the thinnest PPRT (*p* < 0.001).

**Table 2:** Choroid thickness (CT) on different locations

	Average thickness (total)	Emmetropic group (1)	Myopia group (2)	Difference (1–2)	*P* value
Choroid-T	163.21 ± 47.65	181.71 ± 41.83	142.94 ± 45.58	38.76	0.000
Choroid-TS	171.94 ± 43.54	189.67 ± 38.03	152.50 ± 41.01	37.16	0.000
Choroid-S	177.51 ± 47.17	192.16 ± 41.38	161.46 ± 48.17	30.69	0.000
Choroid-NS	178.98 ± 46.05	191.38 ± 43.22	165.39 ± 45.48	25.99	0.000
Choroid-N	180.25 ± 41.78	188.68 ± 39.96	171.01 ± 42.04	17.67	0.009
Choroid-NI	160.54 ± 39.23	171.97 ± 37.25	148.01 ± 37.72	23.96	0.000
Choroid-I	142.88 ± 40.82	156.06 ± 38.37	128.43 ± 38.69	27.62	0.000
Choroid-TI	151.09 ± 42.94	167.83 ± 37.86	132.73 ± 40.82	35.09	0.000
Global PPCT	165.80 ± 39.86	179.93 ± 36.25	150.31 ± 38.04	29.62	0.000
SFCT	302.55 ± 55.95	334.51 ± 25.78	266.02 ± 58.83	68.48	0.000

*T* temporal, *TS* superotemporal, *S* superior, *NS* superonasal, *N* nasal, *NI* inferonasal, *I* inferior, *TI* inferotemporal, *Global PPCT* The average choroidal thickness from the above eight locations, *SFCT* subfoveal choroidal thickness

**Table 3:** Average retinal thickness (RT) at different locations

	Average thickness	Emmetropic group (1)	Myopia group (2)	Difference (1–2)	*P* value
T	71.0 ± 12.65	70.18 ± 11.18	71.93 ± 14.09	−1.74	0.394
TS	129.29 ± 29.96	125.02 ± 27.07	133.91 ± 32.36	−8.89	0.066
S	117.71 ± 32.46	121.02 ± 32.97	114.13 ± 31.73	6.88	0.189
NS	97.64 ± 30.42	106.83 ± 26.61	87.71 ± 31.32	19.12	0.000
N	46.64 ± 11.00	50.06 ± 10.03	42.94 ± 10.86	7.11	0.000
NI	84.41 ± 26.33	90.27 ± 23.61	78.08 ± 27.78	12.19	0.004
I	143.62 ± 38.20	162.51 ± 37.51	123.20 ± 26.88	39.30	0.000
TI	121.40 ± 32.72	111.70 ± 26.29	131.89 ± 35.78	−20.19	0.000
Global PPRT	101.47 ± 10.74	104.70 ± 10.53	97.97 ± 9.89	6.72	0.000
FRT	215.49 ± 42.94	214.20 ± 15.91	216.95 ± 19.37	−2.75	0.341

*T* temporal, *TS* superotemporal, *S* superior, *NS* superonasal, *N* nasal, *NI* inferonasal, *I* inferior, *TI* inferotemporal, *Global PPRT* The average retinal thickness from the above eight locations, *FRT* foveal retinal thickness

The mean global PPRT was 97.97 ± 9.89 μm in the myopic group and 104.70 ± 10.53 μm in the emmetropic group. Significant differences in RT between the myopic group and the emmetropic group were found at all positions except T, TS, S and the fovea. The mean global PPCT was 150.31 ± 38.04 μm in the myopic group and 179.93 ± 36.25 μm in the emmetropic group. Significant differences between the myopic group and the emmetropic group were found at all measured locations (Tables 2 and 3).

SFCT had significantly negative relationship with the fovea RT (*r* = − 0.267, *p* < 0.001), age (*r* = − 0.332, *p* < 0.001) and axial length (*r* = − 0.355, *p* < 0.001). However, the Global PPCT was only significantly associated with axial length (*r* = − 0.254, *p* = 0.002).

## Discussion

**Fig. 2:**
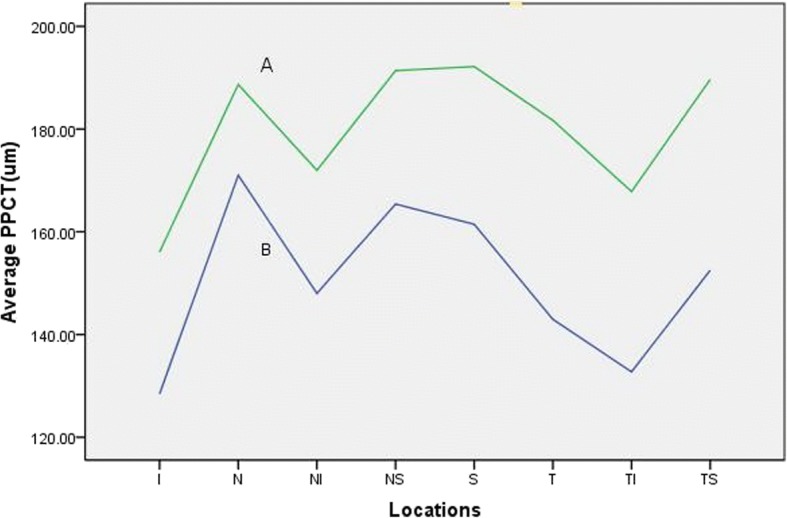
Line charts of average peripapillary choroidal thickness in eight locations with different refractive groups. (A) emmetropic group. (B) myopic group. (I, inferior; IN, inferonasal; IT, inferotemporal; N, nasal; T, temporal; S, superior; SN,superonasal; ST, superotemporal)

In our study, the mean global PPCT was 165.80 ± 39.86 μm and ranged between 77.63 and 263.50 μm. The mean global PPRT was 101.47 ± 10.74 μm and ranged between 77.88 and 125 μm. The peripapillary choroid was thicker nasally and thinner inferiorly while the peripapillary retina was thickest inferiorly and thinnest nasally. The decrease in the global PPCT and SFCT occurred with axial elongation. Significant differences were found between myopia group and emmetropic group in choroidal thickness at all positions, with the myopia group having thinner choroid thickness (Fig. 2).

The peripapillary region of optic disc is of special interest since optic nerve diseases and axial myopia are associated with peripapillary atrophic changes [19]. The mean global PPCT of 165.80 ± 39.86 μm in this study was close to the finding of Wu et al. [17] who measured PPCT with EDI-OCT in 70 children aged 7 to 18y and found that the mean global PPCT was 165.49 ± 33.76 μm. Jiang R [20] once measured peripapillary choroidal thickness of 3060 Chinese participants with an age of 50 years or older and found that mean global PPCT was 134 ± 53 μm. Read et al. [18] measured peripapillary choroidal and retinal thickness of ninety-three children aged between 11 and 16 years and reported an average PPCT of 191 ± 52 μm in the outermost annulus, 77 ± 16 μm in the the innermost annulus. The difference came from the participants, by measuring methods and locations. A relatively large number of children population is needed for further evaluation.

Peripapillary choroidal thickness showed a characteristic distribution pattern, with the thicker PPCT on the nasal position, followed by the superonasal, superior, superotemporal, temporal, inferonasal, inferotemporal and inferior. Inferior was always found to be the thinnest position in both adults and children [16–18, 20]. One explanation was that the optic fissure was located in the inferior aspect of the optic cup, which may be the last part of the globe to close during the ocular development [21]. It was more likely that inferior region would be more susceptible to the hypoxia and elevated IOP, which may cause the thinner choroid [22, 23]. The other indirect evidence was in glaucomatous eyes the superior hemifield was found to be affected more often and more severely than the inferior hemifield [13], which also implied the vulnerability of inferior region. We agreed with the embryological reason and believed that a thinner inferior choroid thickness would be the result of anatomy and function of choroid which may be explained later.

The fovea had the thickest RT and CT among all measured positions. SFCT had a negative relationship with the corresponding retinal thickness. The inferior position had the thickest PPRT but thinnest PPCT among all eight position around the optic disc. This finding may help us understand more about the spatial distribution of retina and choroidal structure. The thickest retina on the fovea was for the best visual resolution, while the thickest choroid on the fovea was to provide nutrients and oxygen to the outer retina so as to get the best central vision [6, 24]. It is recognized that the centre of the optic disc is at about the level of the upper edge of the macula, which makes the inferior of optic disc closer to the fovea [25]. This anatomy character may explain why the peripapillary retina was thickest inferiorly and thinnest nasally.

According to our result, SFCT had negative relationship with the fovea RT and axial length, while the Global PPCT was only significantly associated with axial length. The myopia group had thinner choroid thickness than emmetropic group. Animal experiment showed that the change of axial length produced rapid compensation in choroidal thickness, which may play an important role in emmetropization [17, 26–29]. Our participants were all between 6 and 12 years old, the age bracket of the accomplishment of emmetropization. Therefore, we agreed with the point that choroid might play an important role in the visual regulation of axial growth associated with emmetropization. We also demonstrated the inhomogeneous growth of choroid during the ocular development [6].

Limitations in our study included potential error in manual deliniation of choroidal line and subjective measurements in choroidal thicknesses.

## Conclusions

In conclusion, the peripapillary choroid was thicker nasally and thinner inferiorly, while the peripapillary retina was thickest inferiorly and thinnest nasally. Myopic group had thinner PPCT. The axial length was found to be negatively correlated to PPCT.

## Abbreviations

EDI-OCT: Enhanced-depth imaging optical coherence tomography
FRT: Foveal retinal thickness
PPCT: Peripapillary choroidal thickness
PPRT: Peripapillary retinal thickness
SE: Spherical equivalent refractive error
SFCT: Subfoveal choroidal thickness
